# Report of two cases of influenza virus A/H1N1v and B co-infection during the 2010/2011 epidemics in the Italian Veneto Region

**DOI:** 10.1186/1743-422X-8-502

**Published:** 2011-11-03

**Authors:** Arianna Calistri, Cristiano Salata, Marina Cosentino, Samuele Asnicar, Elisa Franchin, Riccardo Cusinato, Monia Pacenti, Isabella Donatelli, Giorgio Palù

**Affiliations:** 1 Department of Histology, Microbiology and Medical Biotechnologies, Division of Microbiology and Virology, University of Padova, Via A. Gabelli 63, Padova 35121, Italy; 2 Regional Reference Centre for Infectious Diseases, Microbiology and Virology Unit, Hospital of Padova, Via Giustiniani 2, Padova 35128, Italy; 3 Department of Infectious, Parasitic and Immune-mediated Diseases, National Institute of Health (Istituto Superiore di Sanità - ISS), Viale Regina Elena 299, Rome 00161, Italy

**Keywords:** influenza, co-infections, surveillance, diagnostics, dual infections

## Abstract

From October 2010 to April 2011, in the Italian Veneto Region, 1403 hospitalized patients were tested for influenza virus infection by specific real time RT-PCR. Overall, 327 samples were positive for either influenza A (75%) or B (25%) viruses. Among these positive patients two resulted co-infected by A/H1N1v and B viruses. Even though co-infection with both influenza A and B viruses appears to be a rare event, it occurs naturally and may play a role in epidemiology and pathogenicity. In the present study the two co-infected patients were a transplant recipient immunocompromised adult and a child displaying a severe respiratory illness. The co-infection was confirmed by inoculation of the nasopharyngeal swabs in MDCK.2 cells, followed by immunofluorescence and real time RT-PCR assays. Moreover, in the case of the adult patient, the immune system response against both viruses was assayed by hemoagglutination inhibition test against reference influenza virus strains. Both patients fully recovered from infection, without significant differences with mono-infected patients.

## The study

The influenza epidemic in the 2010/2011 season has been characterized in Italy, as in the rest of the world, by a significant co-circulation of influenza A (mostly H1N1v) and influenza B viruses [1]. 1403 nasopharyngeal swabs from hospitalized patients displaying influenza-like illness underwent virological evaluation at the Microbiology and Virology Unit of the Padova Hospital, the Italian Veneto Region reference laboratory for the diagnosis of influenza and other respiratory diseases. In particular, the viral RNA was extracted from the specimens (NucliSENS^® ^easyMAG^®^, Biomerieux, Lyon, France) and a standardized controlled real time Reverse Transcription Polymerase Chain Reaction (RT-PCR) was performed [2]. This method allows the detection of influenza A and B viruses and the analysis of influenza A specific sub-types. In more details, roughly 70 ng of RNA extracted from each sample were assayed with 5 different primer and probe sets specific for influenza A, influenza B, A/H1N1v, A/H3N2 and the housekeeping gene RNasi-P. Overall 327 (23, 3%) samples were positive for influenza virus. Among the positive samples 62 were positive for influenza B virus (25%) and 265 for influenza A virus (75%) with a sharp prevalence of A/H1N1v (183 samples, 69%) over the A/H3N2 (6 samples, 2.2%). It has to be mentioned that 28.8% of influenza A virus positive samples could not be sub-typed, likely due to a low efficiency of the H1N1v specific real time adopted. A similar observation was reported during last influenza season by different European laboratories, adopting this same controlled diagnostic system [1]. The age distribution shows a prevalence of positive patients in the > 60 year old category, as expected during and epidemic season of influenza. Interestingly these data, that are in line with the results reported by different laboratories in the northern hemisphere [1], show a significant co-circulation of influenza A and influenza B viruses, with in general higher prevalence of the A types but with an increase of B type especially between February and March. Co-circulation of multiple strains of influenza viruses and the screenings of several samples by molecular methods provide the unique opportunity for the analysis of simultaneous infections in the human population. Indeed such co-infections have not been widely described and studied so far both because of a poor interest, before the 2009 pandemics, in the laboratory confirmation of suspected influenza virus infections and because of the use of culture-based methods for diagnosis. In our cohort, A/H1N1v and influenza B viral genomes were simultaneously detected in two nasopharyngeal swabs. The viral co-infections were confirmed for both patients by viral isolation in MDCK.2 cells. Specifically the two clinical samples were inoculated in shell vials and influenza A and B immunofluorescence assay (IFA) was performed 24 h post-infection. In addition, a real time RT-PCR assay was performed on culture supernatants (Table 1).

**Table 1:** Analysis performed for the identification of the two double positive samples.

	Nasal swab				MDCK.2 inoculation	
**Patient #**	**real time RT-PCR**		**IFA**		**real time RT-PCR**	
	
	**Type A**	**Type B**	**Type A**	**Type B**	**Type A**	**Type B**

1	+	+	+	+	+	+

2	+	+	+	+	+	+

Nasal swabs of the two co-infected patients were directly analyzed by real time RT-PCR (Nasal swab) and inoculated in MDCK.2 cells (MDCK.2 inoculation) in order to confirm the presence of both viruses (Type A and Type B) by IFA and real time RT-PCR. "+" indicates a positive result.

The first case of dual infection was identified in a kidney transplant recipient adult patient (54 years old), who was not covered by specific vaccination. At the time of virological diagnosis (11^th ^of February, 2011) he displayed mild classical influenza-like illness symptoms. However, due to his state of immunocompromised patient, oseltamivir treatment was started. Interestingly, while both viruses were initially detected at similar concentration, as displayed by the real time cycle threshold (Ct) values, clearance of the B virus was achieved earlier than in the case of influenza A/H1N1v (Table 2). Co-infection with A/H1N1v and B viruses was also confirmed by assaying a patient serum sample collected the 20^th ^of February in an hemoagglutination inhibition (HI) test, performed against the seasonal influenza virus reference strains (A/California/7/2009 H1N1v, A/Perth/16/2009 H3N2 and B/Bangladesh/3333/2007). As expected in an immunocompromised individual, the obtained HI titers were low, 32 and 16 for the virus A/California/7/2009 H1N1v and B/Bangladesh/3333/2007, respectively. No HI titre was detectable in the case of the A/Perth/16/2009 H3N2 virus, employed as specificity control. Taking into account that this patient, as mentioned above, was no covered by influenza vaccination the measured serologic response may be considered a further confirmation of the dual-infection.

**Table 2:** Kinetic of viral clearance in nasal swabs of patient n° 1.

DAYS OF COLLECTION	INFLUENZA	
	
	Type A	Type B
11^th ^February	positive (Ct 31.92)	positive (Ct 31.49)

15^th ^February	positive (Ct 38.40)	negative

18^th ^February	negative	negative

The table reports the results of the real time RT-PCR assays performed on samples collected from patient number 1. Ct stands for real time cycle threshold.

A second case of co-infection by influenza A/H1N1v and B viruses was found in a 6 month old child, displaying severe respiratory symptoms and pneumonia. The 15^th ^of February the child had undertaken antibiotic treatment by intravenous and nebulised routes. The 18^th ^of February the real time RT-PCR assay showed the presence of both influenza virus type B and A, with levels of influenza B virus roughly 10 times higher (Ct 30, 76) with respect to the ones displayed by the A/H1N1v virus (Ct 32, 86). The child did not underwent antiviral treatment, the clinical symptoms and signs resolved quickly and the 22^nd ^of February he was discharged without further virological analysis.

## Discussion

Dual infections of influenza A and B viruses appear to be a rare event and only few publications have reported simultaneous infection by two different types of influenza viruses in humans [3-7]. Thus, the factors that may be responsible for such events are not clear yet, even though the host immune system and the virus properties have been suggested [3-7]. It is interesting to note that both co-infected patients reported in this study represented individuals with a weak immune system, being a kidney transplant recipient and a 6 month year old child, thus suggesting that, indeed, the immunological state of the patient may play an important role in the co-infection establishment. Moreover, the child described in this study represents, to our knowledge, the first report of a dual infected patient with a clinical complication (pneumonia). Indeed, in all publications clinical manifestations in co-infected individuals were identical to those observed in single infections, with classical symptoms, without any clinical complication. However, it has to be mentioned that both these latter observations may be related to the specific cohort (hospitalized patients) taken into consideration in the current study. Thus a clear correlation between dual-infection and immunodeficiency/maturation, or dual infection and severity of the disease cannot be absolutely concluded by these data. On the other hand, both patients fully recovered. An oseltamivir based treatment was adopted only in the case of the adult patient, justified by his immunodeficiency state more than by the severity of the symptoms which remain mild till the viral clearance.

Finally, in contrast with previous literature data [4], in our two cases we did not observe significant differences in the RNA levels of the two viruses in the initial sample, a part for a slight higher amount of influenza B virus in the case of the child (roughly 10 times). However, in the case of the adult patients, for whom three sequential samples were analysed (Table 2), we observed a different kinetic in the viral clearance, with the influenza B virus becoming undetectable more rapidly than the influenza A virus. This finding may related to a different in vivo replication efficiency of two viruses. In fact, influenza A infections are usually more severe than those related to influenza B. However, further studies would be needed to support this hypothesis.

## Competing interests

The authors declare that they have no competing interests.

## Authors' contributions

Conceived and designed the study: AC, CS, ID, GP. Performed analysis: MC, SA, EF. Analysed the data: AC, CS, RC, MP, ID, GP. Wrote the paper: AC, CS. All authors read and approved the final manuscript.
